# Ephenidine: A new psychoactive agent with ketamine-like NMDA receptor antagonist properties

**DOI:** 10.1016/j.neuropharm.2016.08.004

**Published:** 2017-01

**Authors:** Heather Kang, Pojeong Park, Zuner A. Bortolotto, Simon D. Brandt, Tristan Colestock, Jason Wallach, Graham L. Collingridge, David Lodge

**Affiliations:** a Centre for Synaptic Plasticity, School of Clinical Sciences, Dorothy Hodgkin Building, University of Bristol, Bristol, BS1 3NY, UK; b Centre for Synaptic Plasticity, School of Physiology, Pharmacology and Neuroscience, Dorothy Hodgkin Building, University of Bristol, Bristol, BS1 3NY, UK; c School of Pharmacy and Biomolecular Sciences, Liverpool John Moores University, Byrom Street, Liverpool, L3 3AF, UK; d Pharmaceutical Sciences, Philadelphia College of Pharmacy, University of the Sciences, Philadelphia, PA, USA; e Department of Physiology, University of Toronto, Toronto, ON, M5S1A8, Canada; f Lunenfeld-Tanenbaum Research Institute, Mount Sinai Hospital, Toronto, ON, M5G 1X5, Canada

**Keywords:** Ephenidine, Ketamine, NMDA receptor, Dissociative hallucinogen, Legal high, MK-801 binding, Outward rectification, Long-term potentiation, NMDA, *N*-methyl-d-aspartate, AMPA, α-amino-3-hydroxy-5-methyl-4-isoxazolepropionate, D-AP5, D-2-amino-5-phosphonopropionate, LTP, long-term potentiation

## Abstract

To avoid legislation based on chemical structure, research chemicals, frequently used for recreational purposes, are continually being synthesized. *N*-Ethyl-1,2-diphenylethanamine (ephenidine) is a diarylethylamine that has recently become popular with recreational users searching for dissociative hallucinogenic effects.

In the present study, the pharmacological basis of its neural actions has been investigated, initially by assessing its profile in central nervous system receptor binding assays and subsequently in targeted electrophysiological studies. Ephenidine was a potent inhibitor of ^3^H-MK-801 binding (Ki: 66 nM), implying that it acts at the PCP site of the *N*-methyl-d-aspartate (NMDA) receptor. It also showed modest activity at dopamine (379 nM) and noradrenaline (841 nM) transporters and at sigma 1 (629 nM) and sigma 2 (722 nM) binding sites. In experiments of extracellular recording of field excitatory postsynaptic potentials (fEPSPs) from area CA1 of rat hippocampal slices, ephenidine, 1 and 10 μM, respectively, produced a 25% and a near maximal inhibition of the NMDA receptor mediated fEPSP after 4 h superfusion. By contrast, ephenidine (50 μM) did not affect the AMPA receptor mediated fEPSPs. In whole cell patch clamp recordings, from hippocampal pyramidal cells, ephenidine (10 μM) blocked NMDA receptor-mediated EPSCs in a highly voltage-dependent manner. Additionally, ephenidine, 10 μM, blocked the induction of long term potentiation (LTP) in CA1 induced by theta burst stimulation.

The present data show that the new psychoactive substance, ephenidine, is a selective NMDA receptor antagonist with a voltage-dependent profile similar to ketamine. Such properties help explain the dissociative, cognitive and hallucinogenic effects in man.

This article is part of the Special Issue entitled ‘Ionotropic glutamate receptors’.

## Highlights

• New ‘legal high’, the dissociative ephenidine, displaces MK-801 binding.
• Ephenidine, like ketamine, blocks NMDA receptor mediated synaptic potentials and plasticity.
• Ephenidine, like ketamine, blocks the NMDA receptor in a highly voltage-dependent manner.
• Ephenidine blocks long-term potentiation, LTP.
• NMDA receptor antagonism likely underlies the psychoactive effects of ephenidine.

## 1 Introduction

Shortly after their development as potential general anesthetics for veterinary and human use (Greifenstein et al., 1958, McCarthy et al., 1965, Domino et al., 1965), both phencyclidine (PCP) and ketamine were widely abused throughout the world for their dissociative effects (Petersen and Stillman, 1978, Jansen, 2000). Although PCP is still abused as a ‘street drug’ in the USA, its misuse has been reduced particularly in Europe because of severe and long lasting psychotomimetic effects, including lethality (Moeller et al., 2008) whereas the shorter-acting ketamine has remained a popular recreational drug (Freese et al., 2002, Nutt et al., 2007, Morris and Wallach, 2014), although not without dangers (Morgan and Curran, 2012). However, legislation has been enacted in many countries in an attempt to prevent their use and sale, which in turn has resulted in a burgeoning of new chemicals with dissociative properties (Roth et al., 2013, Morris and Wallach, 2014). Interestingly, the most common structures, like phencyclidine, are tricyclic compounds and include various 1,2-diarylethylamines e.g. diphenidine and 2-methoxydiphenidine (Morris and Wallach, 2014). Such compounds, although structurally distinct from arylcyclohexylamines, like PCP and ketamine, are well documented in on-line anecdotal reports, as having potent and long lasting dissociative effects in man (http://www.bluelight.org/vb/threads/668291-The-Big-amp-Dandy-Diphenidine-Thread; http://www.erowid.org/chemicals/methoxphenidine/methoxphenidine_timeline.php; http://drugs-forum.com/forum/showthread.php?t=273812). Like the original dissociative anesthetics (Anis et al., 1983) and other dissociative hallucinogens (Lodge and Mercier, 2015), these tricyclic 1,2-diarylethylamines have proved to be potent and selective NMDA antagonists (Wallach et al., 2016).

Recently, ephenidine, a two ringed *N*-ethyl-1,2-diphenylethylamine, has become available and anecdotally appears popular with users of dissociative research chemicals e.g. ‘finally a worthy alternative to ketamine … ’, (http://www.bluelight.org/vb/threads/766110-The-Big-amp-Dandy-Ephenidine-%3F28N-ethyl-1-2-diphenylethylamine%3F29-Thread; http://www.psychonaut.com/sintetici/56569-ephenidine.html). An early brief medicinal chemistry report, without detailing synthesis, suggested that ephenidine displaced PCP binding (Thurkauf et al., 1989). However, no suggestion of the relationship to NMDA receptor antagonism was made nor were its selectivity, its mode of action and its potential to affect synaptic function and plasticity explored. We have therefore addressed these and further compared the effects of ephenidine with those of ketamine on synaptic transmission in hippocampal brain slices using both extracellular and whole-cell recording techniques. We have also examined the selectivity of ephenidine by comparing its potency at displacing MK-801 binding with its actions on a wide range of CNS receptors.

The data show that ephenidine is a relatively selective, voltage-dependent NMDA antagonist that potently blocks LTP. These observations can explain the psychotomimetic effects of ephenidine and predict a range of side-effects including memory impairments.

## 2 Methods

### 2.1 Preparation of ephenidine

Full details of the synthesis and analytical characterization of ephenidine (*N*-ethyl-1,2-diphenylethylamine) are given in Supplement 1.

### 2.2 Receptor binding experiments

The binding affinity (K_i_) of ephenidine to the MK-801 binding site of the NMDA receptor was determined as described by Sharma and Reynolds (1999). Briefly, after thorough washing of the homogenate of whole rat brain (Pel-Freez Biologicals), suspensions in 10 mM HEPES (pH 7.4 at room temperature), containing 100 μg/mL protein, were incubated in the dark on a mechanical rocker for 2 h in the presence of 1 nM (+)-[^3^H]-MK-801, 100 μM glutamate, 10 μM glycine, and various concentrations of ephenidine, ketamine and MK-801 or 30 μM (+)-MK-801 for nonspecific binding (Sharma and Reynolds, 1999). Termination of reaction was performed via vacuum filtration using a 24 well cell harvester (Brandel, Gaithersburg, MD) over presoaked GF/B glass fiber filters (Brandel, Gaithersburg, MD). Filters were washed with room temperature assay buffer (3 × 5 mL). Trapped tritium was measured via liquid scintillation counting, using a Beckman LS 6500 multipurpose scintillation counter (BeckmanCoulter, USA) at 57% efficiency. IC_50_ values were determined in Graphpad Prism 5.0 using non-linear regression with log-concentration plotted against percent specific binding. Percent specific binding for [^3^H]-MK-801 in control experiment was ∼95% of total. K_i_ values were calculated using the equation of Cheng and Prusoff (1973). The K_d_ for (+)-MK-801, 1.75 nM, was determined via homologous binding assay as described by Sharma and Reynolds (1999) and is consistent with the literature. Protein concentration was determined via the Bradford method using Coomassie protein assay reagent (Sigma, USA) with rat albumin (Sigma, USA) as standard. Experiments were performed in duplicate and repeated three or four times.

Displacement by ephenidine in binding assays of a further 45 CNS receptors was performed through the National Institute of Mental Health Psychoactive Drug Screening Program (NIMH PDSP). Briefly ephenidine, dissolved in DMSO, was subject to a primary screen at a concentration of 10 μM (see Suppl. 2 for radioligands used). Compounds exhibiting >50% inhibition were subjected to a secondary assay at varying concentrations to determine K_i_ values. Additional experimental details are available in the NIMH PDSP protocol book (Roth et al., 2013).

### 2.3 Electrophysiology in hippocampal slices

Male Wistar and Sprague-Dawley rats (Crl:Wi; Charles River, UK) aged 3–10 weeks old were killed by dislocation of cervical vertebrae according to Schedule 1 of the United Kingdom (Scientific Procedures) Act of 1986. Brains were rapidly removed and put in artificial cerebrospinal fluid (aCSF) consisting of 124 mM NaCl, 26 mM NaHCO_3_, 3 mM KCl, 1.4 mM NaH_2_PO4, 1 mM MgSO_4_, 2 mM CaCl_2_, and 10 mM d-glucose and continuously oxygenated with 95% O_2_ and 5% CO_2_. Parasagittal brain slices, 400 μm thick, of the hippocampus were removed, stored and placed in a submerged recording chamber at 28–30 °C.

**Fig. 1:**
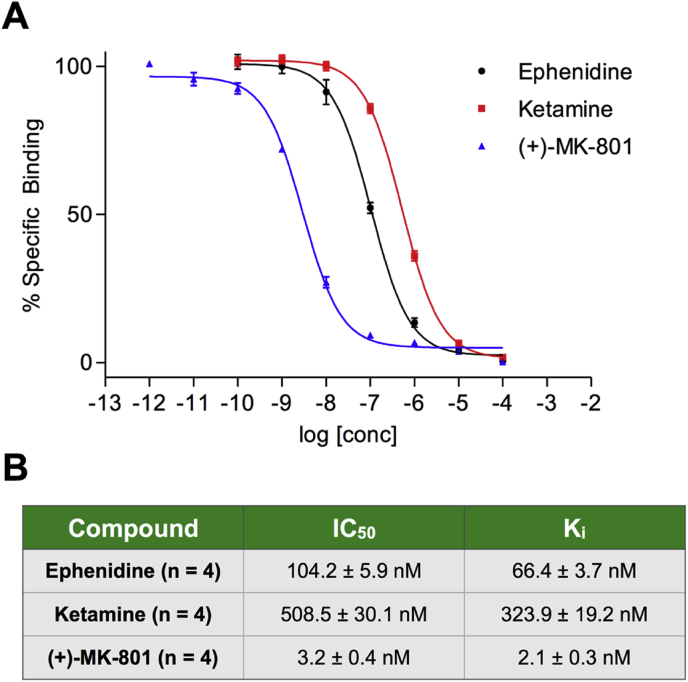
^3^H-MK-801 binding experiments. **A**. Displacement curves showing the concentration-response relationship of ephenidine, ketamine (data from Wallach et al., 2016) and MK-801. **B**. Table showing the calculated IC_50_ and K_i_ values for the 3 competing ligands.

**Fig. 2:**
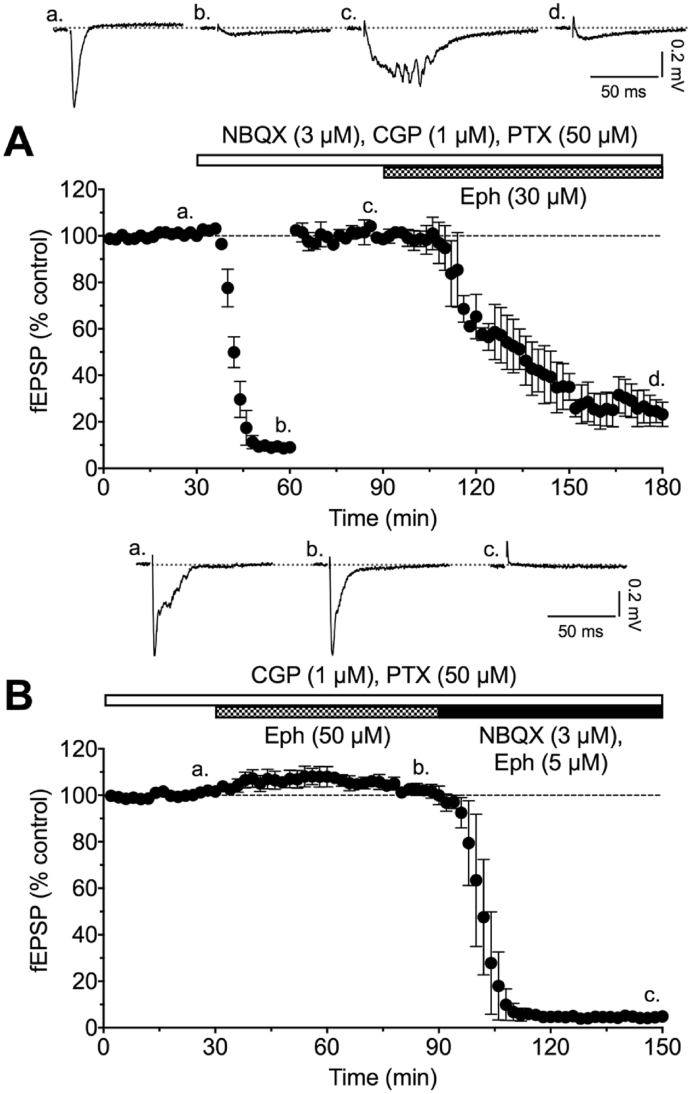
Graphs showing time-course of block of the NMDA receptor-mediated fEPSPs in CA1 of hippocampal slices by 30 μM ephenidine **(A)** and lack of effect of ephenidine 50 μM on AMPA receptor-mediated fEPSPs **(B)**. The bars above the graphs indicate the superfusion times for various compounds. The sample traces at the top show typical responses at the times indicated by the associated lower case letters below.

Recordings of extracellular synaptic activity were made (Ceolin et al., 2012, Volianskis et al., 2013) using a bipolar electrode to deliver stimuli (0.03 Hz) to the Schaffer collateral pathway to evoke field excitatory postsynaptic potentials (fEPSPs) recorded from a glass microelectrode (3–6 MΩ) in the stratum radiatum of area CA1. The NMDA receptor-mediated component of the fEPSP (NMDA-fEPSP) was isolated by adding 3 μM NBQX, 50 μM picrotoxin and 1 μM CGP 55845 to the aCSF, which abolished AMPA and GABA receptor mediated transmission. In initial experiments after 30 min of stable control responses, the remaining response was challenged with 30 μM ephenidine. Using this protocol for isolating NMDA-fEPSPs (see Fig. 2A), 1 and 10 μM ephenidine or 1 and 10 μM D-2-amino-5-phosphonopentanoic acid (D-AP5) were added to the perfusate for up to 4 h while recording the amplitude and area of the NMDA-fEPSP on-line using WinLTP (Anderson and Collingridge, 2007). Single exponential curves were fitted to show likely half-times and minimum plateau responses achieved with each concentration used. For selectivity experiments, 50 μM picrotoxin and 1 μM CGP 55845 were added to the solution from the beginning of the experiment and 50 μM ephenidine was superfused for 60 min in the absence of other pharmacological agent. Under these conditions the AMPA receptor mediated fEPSP dominates the recording. 5 μM ephenidine with 3 μM NBQX were then added to the perfusate for another 1 h to confirm that NMDA-fEPSP had been abolished.

To study the effect of ephenidine on long-term potentiation (LTP), slices were incubated in control aCSF or aCSF containing 10 μM ephenidine for at least 2 h before transferring to the recording chamber, fEPSPs were recorded as above without addition of other agents. After obtaining stable responses, a theta burst stimulation (TBS; Park et al., 2016) was applied to the Schaffer collateral pathway and the amplitude of the fEPSP in CA1 monitored for at least a further 40 min.

Whole-cell patch clamp recordings of excitatory postsynaptic currents (EPSCs) were made from CA1 pyramidal cells under visual guidance with IR-DIC optics as described by Park et al. (2016). The recording chamber was maintained at 32 °C. Borosilicate glass pipettes were used with a resistance of 3–5 MΩ. The internal solution comprised (mM): 8 NaCl, 130 CsMeSO_3_, 10 HEPES, 0.5 EGTA, 4 Mg-ATP, 0.3 Na_3_-GTP, 5 QX-314 and 0.1 spermine. The pH was adjusted to 7.2–7.3 with CsOH and osmolarity to 285–290 mOsm. Peak amplitude of EPSCs was measured while varying holding voltage from −60 mV to +60 mV and stimulating the Schaffer collateral pathway at a frequency of 0.1 Hz at constant intensity. Five consecutive responses obtained at each holding potential were averaged to plot the current-voltage relationship. Currents through NMDA receptors were pharmacologically isolated using 10 μM NBQX, 50 μM picrotoxin and 20 μM (+)-bicuculline. In initial experiments, MgSO_4_ was omitted from the bath solution for at least 30 min before patching and replaced with 30 μM ephenidine, 10 μM ketamine, 30 μM D-AP5 or 2 mM MgSO_4_. Rectification indices were calculated from the EPSC values at +40 mV and −40 mV. In subsequent experiments, slices were incubated in aCSF with no added MgSO_4_, before transferring to the recording chamber to obtain control current-voltage plots from NMDA-EPSCs as above before adding 2 mM MgSO_4_ or 30 μM ephenidine for 30 min and repeating the current-voltage protocol. Representative sample traces are shown from typical experiments, and stimulus artifacts blanked for clarity. Data were normalized to the baseline preceding drug application, and are presented as mean ± SEM and compared using one-way ANOVA and level of significance is denoted as *** for p values < 0.001.

## 3 Results

### 3.1 Receptor binding data

Fig. 1 shows the (+)-[^3^H]-MK-801 displacement curves by concentrations of ephenidine, ketamine and MK-801 from 0.1 nM to 100 μM. Thus ephenidine showed nanomolar affinity with a calculated K_i_ value of 66.4 ± 3.7 nM. This compares with Ki values in the same assay for ketamine and MK-801 of 324 ± 19 and 2.1 ± 0.3 nM respectively (Wallach et al., 2016). The parallel nature of the curves suggests that all three compounds bind to the same site in the NMDA receptor-channel complex.

The data from the NIMH PDSP assay (Table S1) shows that ephenidine has submicromolar affinity for the dopamine (379 nM) and noradrenaline (841 nM) transporters and for sigma 1 (628 nM) and sigma 2 (721 nM) binding sites. The remaining 41 receptors evaluated in the binding assays (see Supplement 2) showed less than 50% displacement with 10 μM ephenidine.

### 3.2 Electrophysiology in hippocampal slices

**Fig. 3:**
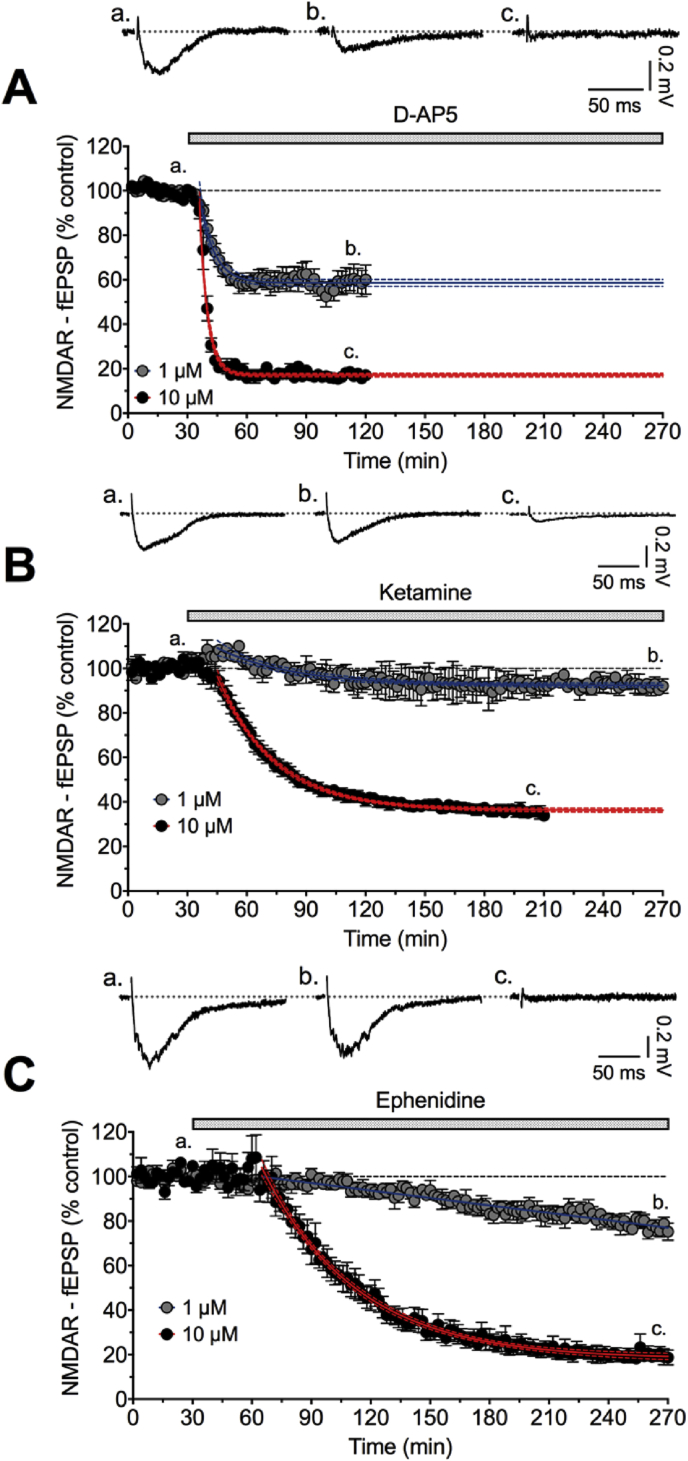
Time-course of inhibition of NMDA receptor-mediated fEPSP by 1 and 10 μM D-AP5 **(A)**, ketamine **(B)** and ephenidine **(C)**. In each graph which is the average of 3–5 experiments, a baseline of 30 min was obtained before superfusing the hippocampal slices with indicated compound. Typical examples of raw data (**a**, **b** and **c**) are taken at the times indicated on the graphs below.

In initial experiments, superfusion of hippocampal slices with NBQX, picrotoxin and CGP55845 containing aCSF to reduce AMPA, GABA-A and GABA-B receptor-mediated events resulted in fEPSPs, which were slowly reduced by 30 μM ephenidine (Fig 2A). The rapid reduction of such fEPSPs in the presence of 1 and 10 μM D-AP5 (Fig 3A) and slower reduction in presence of 1 and 10 μM ketamine (Fig 3B) confirms their dependence on NMDA receptors. To investigate further the sensitivity of the fEPSP to ephenidine, in the presence of the same cocktail of GABA and AMPA receptor antagonists, it was superfused at 10 μM, which slowly reduced the fEPSP reaching a near maximal effect after 4 h. By contrast, the effect of 1 μM ephenidine on this fEPSP was limited to approximate 25% inhibition after 4 h perfusion (Fig 3C). This slow antagonism by ephenidine is typical of other use-dependent uncompetitive NMDA receptor antagonists, e.g. ketamine (Fig 3B) and suggests a similar mode of action (see Wallach et al., 2016).

To study the selectivity of ephenidine against the AMPA subtype of glutamate receptor, the effect of ephenidine was studied on the early component of the fEPSP in the absence of NBQX (Fig 2B). One hour of superfusion with 50 μM ephenidine produced no significant reduction of the peak amplitude of the fEPSP, although it can be seen from the individual recordings that the late NMDA receptor-mediated component is considerably reduced (Fig 2B). Switching the superfusion to one containing 3 μM NBQX resulted in a rapid block of the remaining fEPSP (Fig 2B), demonstrating its dependence on AMPA receptors.

**Fig. 4:**
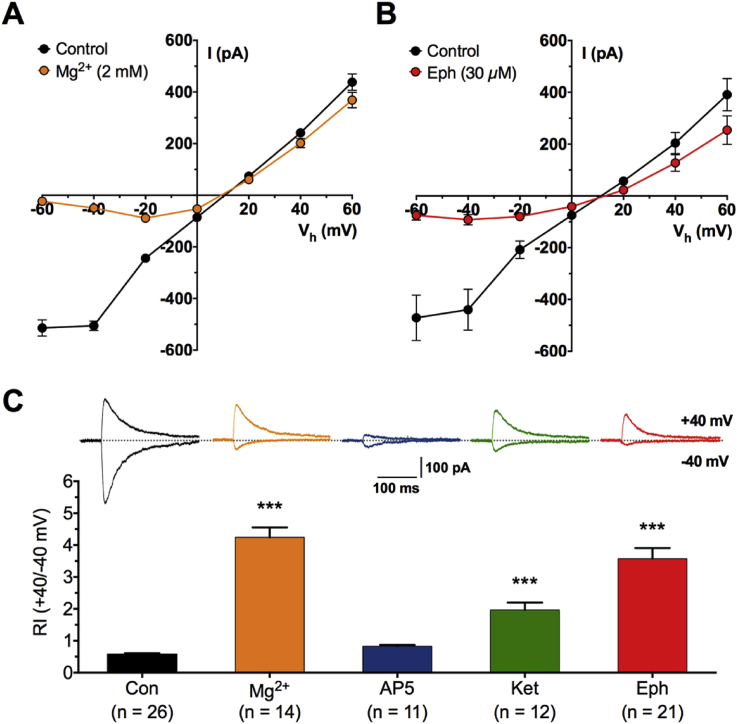
Whole cell patch clamp recordings of EPSCs from hippocampal pyramidal neurones. **A**. Current-voltage plots for peak EPSCs before and 30 min after addition of 2 mM Mg^2+^ ions to the aCSF as indicated by the coloured symbols (n = 7). **B**. As for **(A)** above, no added Mg^2+^ ions but with the addition of 30 μM ephenidine (n = 6). **C**. Bar chart showing rectification indices (RI; +40mV/-40 mV) for EPSCs recorded in aCSF with no added Mg^2+^ (n = 26; black), 2 mM Mg^2+^ (n = 14; orange), 30 μM D-AP5 (n = 11; blue), 10 μM ketamine (n = 12; green) or 30 μM ephenidine (n = 21; red). Examples of EPSCs recorded at +40 mV and −40 mV in the various conditions are shown above each bar graph. (For interpretation of the references to colour in this figure legend, the reader is referred to the web version of this article.)

To compare the effect of ephenidine on current-voltage relationship of NMDA receptor mediated synaptic currents, patch clamp recordings were made in the presence of the above cocktail of drugs and in the absence of added Mg^2+^ ions before adding 2 mM Mg^2+^ (Fig 4A) or 30 μM ephenidine (Fig 4B) to the aCSF for 30 min. Ephenidine showed a profile typical of a channel blocker, i.e. reduction of inward current at negative holding potentials. The rectification index (RI), expressed as the ratio of currents at holding potentials of +40 mV to −40 mV, was also used to quantify this profile. In separate studies where slices were bathed in drug-containing aCSF before patch clamping, RIs were calculated from similar current-voltage measurements. For example, at −40 mV, the mean current in 30 μM ephenidine was −48 ± 8 pA and at +40 mV was 133 ± 12 pA. The mean of the individual RI values was 3.6 ± 0.3, which compares with an RI of 0.6 ± 0.01 at the same holding potentials in control aCSF with no added Mg^2+^ ions (Fig 4C). Thus, it appears from the RI values (Fig 4C) that 30 μM ephenidine falls between 2 mM Mg^2+^ (RI = 4.2 ± 0.3) and 10 μM ketamine (RI = 2.0 ± 0.2). This is in contrast to the competitive antagonist, D-AP5, which, as expected, reduced both inward and outward current, so that its RI (0.8 ± 0.01) remains similar to the Mg^2+^-free control value (Fig 4C).

**Fig. 5:**
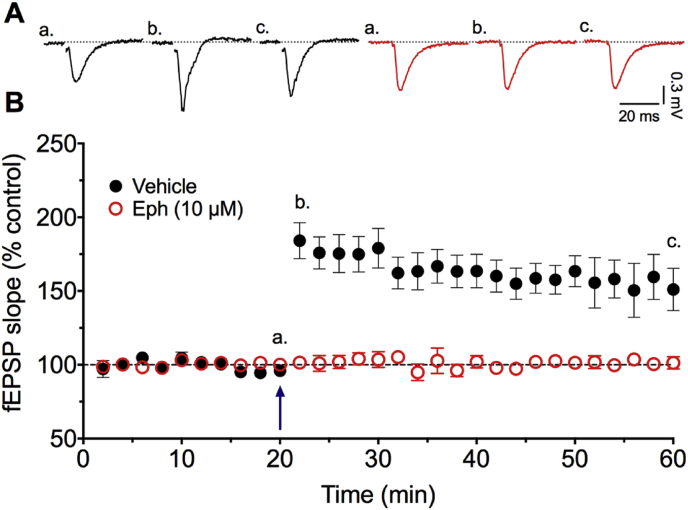
Effect of ephenidine on LTP of fEPSPs in the Schaffer collateral CA1 pathway. **A**. Examples of the fEPSPs before **(a)**, 5 min after **(b)** and 40 min after **(c)** theta burst stimulation (TBS) of the input to CA1 neurones in the absence (black) or presence (red) of 10 μM ephenidine. **B**. Graph showing the mean fEPSP slope values from 5 experiments on hippocampal slices under control conditions and from 5 interleaved experiments on slices incubated in 10 μM ephenidine. TBS is indicated by the arrow at 20 min. (For interpretation of the references to colour in this figure legend, the reader is referred to the web version of this article.)

Other NMDA antagonists block the form of synaptic plasticity known as long-term potentiation (LTP), although interestingly this ability varies according to the nature of different channel blockers (Frankiewicz et al., 1996). We therefore tested ephenidine in a standard LTP protocol. Theta burst stimulation (TBS) applied to the Schaffer collateral pathway provided a robust potentiation of the fEPSP slope (Fig 5) in control slices interleaved between slices incubated with 30 (n = 3; data not shown) or 10 μM (n = 5; Fig 5) ephenidine. At both concentrations, ephenidine completely blocked the induction of LTP.

## 4 Discussion

We have shown that ephenidine, an abused psychoactive substance (Brandt et al., 2014) with dissociative effects in man, is a relatively selective, potent and voltage-dependent NMDA receptor antagonist and hence can be classified as an uncompetitive channel blocking compound.

Although ephenidine's use was detected in Germany in 2008 (Westphal et al., 2010), its availability from internet retailers has only been a more recent phenomenon. Ephenidine appears in older chemical (Goodson et al., 1946) and pharmacological (Tainter et al., 1943) literature. In a brief medicinal chemistry report, relating it to MK-801 as an anticonvulsant, its affinity for the PCP binding site was given as 257 nM (Thurkauf et al., 1989) and information regarding its rapid metabolism has also recently appeared (Wink et al., 2014, Wink et al., 2015). However, its ability to affect NMDA receptor function had not been explored.

The voltage-dependent effects, we report here for ephenidine (Fig 4), are similar to earlier reports on ketamine (MacDonald et al., 1987, Davies et al., 1988a, Davies et al., 1988b), memantine and MK-801 (Wong et al., 1986, Chen et al., 1992, Frankiewicz et al., 1996) and Mg^2+^ ions (Nowak et al., 1984, Mayer et al., 1984), in which outward currents were the less affected. Interestingly the nature of the voltage-dependency block of NMDA receptors has been related to therapeutic potential (Frankiewicz et al., 1996). In the present experiments, due to slow wash-in times of ephenidine, it was not possible to achieve a full equilibrium block. However, ephenidine is clearly a highly voltage-dependent blocker of synaptic NMDA receptors and has a potency slightly greater than ketamine at resting membrane potentials. When compared with other uncompetitive NMDA antagonists in the same protocol for studying NMDA-fEPSPs (Wallach et al., 2016), the potency and pharmacodynamic properties of ephenidine are closer to those of ketamine than of phencyclidine.

Perhaps because of these pharmacodynamic properties or for some pharmacokinetic reason, ephenidine appears anecdotally to be popular amongst those interested in the dissociative state (see Introduction). The subjective effects reported appear dose-dependent and include dissociative-like effects including mood and thought alteration and complex visual hallucinations at doses between 100 and 500 mg (see Introduction).

Many voltage-dependent uncompetitive NMDA antagonists, such as ketamine, PCP and dextromethorphan, also show schizophrenia-like effects in man (Lodge and Mercier, 2015). Although ephenidine is not described as a psychotomimetic agent in the scientific literature, anecdotal descriptions of the dissociative effects of ephenidine (see above) suggest that, in high enough doses, it mimics some of the symptoms of schizophrenia. Casual users of ephenidine may put themselves in danger particularly as a result of the dissociative and analgesic effects of the NMDA receptor antagonism, as has been similarly described for ketamine abuse (Morgan and Curran, 2012). The block of the induction of LTP with 10 μM ephenidine predicts cognitive disruption and amnesia (Bliss and Collingridge, 1993). Our data also suggests that at even higher doses, interaction with monoamine transporters and sigma receptors may contribute to the behavioural effects of ephenidine.

Beside the analgesic and neuroprotective properties of ketamine (see Lodge and Mercier, 2015), an exciting novel therapeutic target for ketamine has emerged. Thus, ketamine and some other NMDA receptor antagonists have recently been proposed as rapidly acting anti-depressants (Berman et al., 2000, Zarate et al., 2006, Price et al., 2009). During depressed mood and during stress, memories of unpleasant events may be established through long-term potentiation (LTP) and long-term depression (LTD). Treatment with NMDA receptor antagonists, inhibitors of LTP and LTD, may block this cycle of pathological plasticity and change mood (Collingridge et al., 2010). Not all NMDA receptor antagonists have this property. Thus MK-801, another channel blocker with slower pharmacodynamic and pharmacokinetic properties than ketamine (see Wallach et al., 2016), does not show sustained anti-depressant-like effects (Maeng et al., 2008). It remains to be seen whether ephenidine, with pharmacodynamic and LTP blocking properties similar to ketamine, will have the appropriate profile for such a therapeutic indication.
