# Supplementary material for: Ephenidine: A new psychoactive agent with ketamine-like NMDA receptor antagonist properties
Source: Neuropharmacology. 2017 Jan;112(Pt A):144–9. doi: 10.1016/j.neuropharm.2016.08.004 (PMC5084681; doi:10.1016/j.neuropharm.2016.08.004)
Supplement: Supplementary file 1 [file mmc1.docx]

**SUPPLEMENT 1. Preparation of Ephenidine**

**Materials**

All starting materials, reagents and solvents for synthesis (≥ 95%) were obtained from Sigma-Aldrich (St. Louis, USA). Column chromatography was conducted using Merck silica gel, grade 9385 (230-400 mesh, 60 Å). Melting point ranges were obtained with a DigiMelt A160 SRS melting point apparatus (Stanford Research Systems, Sunnyvale, USA) at a ramp rate of 2 °C/min and are uncorrected.

**Instrumentation**

*Gas chromatography ion trap mass spectrometry*

Chemical ionization mass spectra were recorded using HPLC grade methanol as the liquid CI reagent. A Varian 450-GC gas chromatograph coupled to a Varian 220-MS ion trap mass spectrometer and a Varian 8400 autosampler was employed with a Varian CP-1177 injector (275 ºC) in split mode (1:50) (Walnut Creek, CA, USA). The Varian MS Data Review function of the Workstation software, version 6.91, was used for data acquisition. The carrier gas was helium at a flow rate of 1 mL/min using the EFC constant flow mode. The default settings for CI ionization parameters (0.4 s/scan) were used: CI storage level *m/z* 19.0; ejection amplitude *m/z* 15.0; background mass *m/z* 55; maximum ionization time 2000 µs; maximum reaction time 40 ms; target TIC 5000 counts. Temperatures for ion trap, manifold, and transfer line were set at 170 °C, 120 °C, and 280 °C, respectively. An Agilent J&W VF-5ms GC column (30 m × 0.25 mm, 0.25 µm) was employed for separation. The starting temperature was set at 80 ºC and held for 1 min. The temperature then increased at 20 ºC/min to 280 ºC and held constant for 9 min to give a total run time of 20.00 min.

Atmospheric Solids Analysis Probe Mass spectrometry

Spectra was recorded on an Advion expression^S^ CMS (New York, USA) using an atmospheric solids analysis probe (ASAP) source from Advion*.* Nitrogen was used as the source gas. Capillary temperature was set at 150 ºC, with the capillary voltage of 120 V, source voltage off-set was set to 20 and source voltage span to 0. Source gas temperature was 200 ºC and APCI corona discharge set to 5 A.

High Resolution

High resolution mass spectrometry was run using an atmospheric pressure solids analysis probe (ASAP) on a Thermo Fisher Scientific Inc., Waltham, MA, USA) Orbitrap Exactive with an Ion Max source in positive mode. Measured accurate masses were within ± 5 ppm of the theoretical masses. The following parameters were used: resolution was set to ultra high, sheath gas (N_2_) flow 5 (arbitrary units), auxiliary gas flow 6 (arbitrary units), sweep gas flow 0 (arbitarty units), corona discharge 4 kV, capillary temperature 275 °C, capillary voltage 25.0 V, skimmer voltage 14 V and a tube lens voltage of 85 V.

Analytical data image

Figure S1/1. High resolution ASAP mass spectrometry.


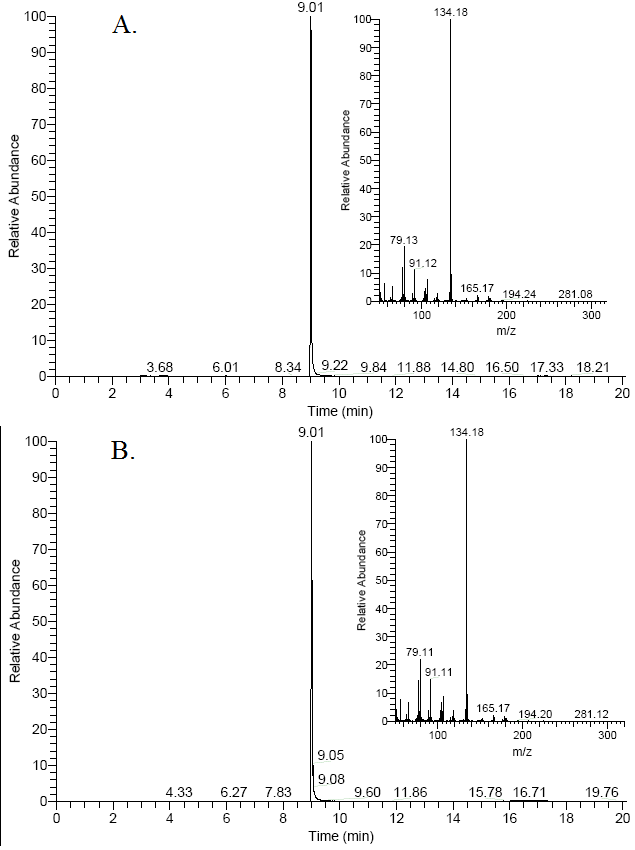


Figure S1/2. Ephenidine GC/MS spectra. A. Synthesized standard of ephenidine HCl. B. test purchase of ephenidine ‘research chemical’.

*Nuclear magnetic resonance spectroscopy*

^1^H (400 MHz) and ^13^C NMR PENDANT spectra (100 MHz) was obtained on the hydrochloride salt in CDCl_3_ (100% and 99.96% D, 0.03% (v/v) TMS) at a concentration of 20 mg/mL on a Bruker Ultrashield 400 plus spectrometer with a 5 mm BBO S1 (Z gradient plus) probe at 24 °C.  Internal chemical shift references were TMS (δ = 0.00 ppm) and solvent (δ = 77.0 ppm). Assignments were made using chemical shift splitting and position and 2-D heteronuclear experiments (HMQC and HMBC).

**Synthesis**

*Synthesis of N-ethyl-1,2-diphenylethan-1-amine*

1,2-Diphenylethylamine (500 mg, 2.5 mmol) was dissolved in 2 mL trimethylamine. To this was slowly added 270 uL (3.8 mmol) acetyl chloride drop wise while vigorously mixing the solution with a metal spatula. The initial golden liquid quickly set to a white solid mass. This mass was mixed vigorously, allowed to sit overnight and then suspended between 30 mL 1N aqueous HCl and 30 mL ethyl acetate. The organic phase was separated, the aqueous extracted once more with 30 mL ethyl acetate and the organic phases pooled, washed with 1N aqueous HCl (2 x 50 mL), dilute KOH solution (1 x 50 mL), distilled water (1 x 50 mL), saline (1 x 10 mL), dried over sodium sulfate and solvents evaporated under vacuum to give a white solid. Solids were crystallized by dissolving in 10 mL boiling ethyl acetate. 20 mL of hexanes were added and the solution allowed to sit at room temperature for several days. The resulting white fluffy needles were collected by decanting and were washed with a small volume of hexanes and aired dried to give 250 mg. mp: 151.0-152.5 C.

*N*-acetyl-1,2-diphenylethylamine ((0.42 mmol, 100 mg) was dissolved in 3 mL dry THF. While stirring under argon, 375 uL of vitride ((1.25 mmol, 65% solution in toluene) was slowly added dropwise. The reaction was heated in a CEM discover microwave reactor at 75 C for 35 minutes (20 psi, 50 W). TLC showed the reaction to be complete. The reaction was then quenched by the cautious addition to distilled H2O. KOH solution was added until basic and the solution was extracted with ethyl acetate (3 x 20 mL). The pooled organic phases were extracted with 0.25 N aqueous HCl (3 x 50 mL). The extracts were pooled, made basic with KOH pellets and extracted with ethyl acetate (3 x 30 mL). The pooled organic extracts were then washed with saline, dried with sodium sulfate, and evaporated under vacuum to give 50 mg of a colorless oil. This material was purified via flash column chromatography on silica gel with hexanes : ethyl acetate (4:1) with 0.5% trimethylamine. Desired fractions (MS) were pooled and evaporated to give 35 mg of the product as a colorless oil.

HCl salt: The freebase was dissolved in 20 mL ethanol (200 proof) and titrated to ~ pH 1 with concentrated HCl. Solvents were evaporated. Acetone was added and evaporation continued, this was repeated until all residual moisture and acid were driven off. The resulting white solids were crystallized by dissolving in 3 mL methanol and diluting with 20 mL diethyl ether and allowed to sit at room temperature for several hours. The solvent was decanted, crystals washed with diethyl ether and ethyl acetate and dried in an oven (60 °C) to five fluffy white needles. Mp: 241.0-241.5 C.

ASAP MS: 227.22 (15), 226.22 (100) [M+H]^+^, 181.16 (55) [M-NCH_2_CH_3_]^+^

High-resolution ASAP MS: Calculated: 226.1580. Predicted for C_16_H_20_N: 226.1590

^1^H NMR (400 MHz, CDCl_3_): 10.51 (s, 1 NH^+^), 10.13 (s, 1 NH^+^), 7.52-7.41 m (2’’, 6’’, 2 Ar-H), 7.40-7.28 m (3’, 5’, 4’’, 3 Ar-H), 7.16-7.04 m (3’’-5’’, 4’, 3 Ar-H), 6.97-6.84 m (2’, 6’, 2 Ar-H), 4.26-4.14 m (C_1_, 1H), 4.01 dd (*J* = 12.7, 3.5 Hz, C_2_, 1H), 3.48 t (*J* = 12.2 Hz, C_2_, 1H), 2.95-2.74 m (NCH_2_CH_3_, 2H), 1.48 t (CH_3_, 3H)

^13^C NMR (100 MHz, CDCl_3_): 135.62 (C_1’’_, 1 Ar-H), 133.50 (C_1’_, 1 Ar-H), 129.44 (2’, 6’, 2 Ar-H), 129.25 (4’’, 1 Ar-H), 129.16 (3’’, 5’’, 2 Ar-H), 128.58 (2’’, 6’’, 2 Ar-H), 128.26 (3’, 5’, 2 Ar-H), 126.73 (4’, 1 Ar-H), 64.93 (C_1_), 41.09 (NCH_2_CH_3_), 40.78 (C_2_), 11.28 (CH_3_)

**SUPPLEMENT 2. Psychoactive Drug Screening Program (NIMH, PDSP).**

**Table S2/1.** *Radioligand and concentrations used for the NIMH PDSP receptor binding screening assays*.

| Receptor | Radioligand  (concentration) | Receptor | Radioligand  (concentration) |
| --- | --- | --- | --- |
| 5-HT1A | [^3^H]8-OH-DPAT (0.5 nM) | H3 | [^3^H]-alpha-methylhistamine (0.4 nM) |
| 5-HT1B | [^3^H]GR127543 (0.3 nM) | H4 | [^3^H]Histamine (5 nM) |
| 5-HT1D | [^3^H]GR127543 (0.3 nM) | SERT | [^3^H]Citalopram (0.5 nM) |
| 5-HT1E | [^3^H]5-HT (3 nM) | NET | [^3^H]Nisoxetine (0.5 nM) |
| 5-HT2A | [^3^H]Ketanserin (0.5 nM) | DAT | [^3^H]WIN35428 (0.5 nM) |
| 5-HT2B | [^3^H]LSD (1 nM) | BZP | [^3^H]Flunitrazepam (0.5 nM) |
| 5-HT2C | [^3^H]Mesulergine (0.5 nM) | Alpha1A | [^3^H]Prazosin (0.7 nM) |
| 5-HT3 | [^3^H]LY278584 (0.3 nM) | Alpha1B | [^3^H]Prazosin (0.7 nM) |
| 5-HT5a | [^3^H]LSD (1 nM) | Alpha2A | [^3^H]Clonidine (1 nM) |
| 5-HT6 | [^3^H]LSD (1 nM) | Alpha2B | [^3^H]Clonidine (1 nM) |
| 5-HT7 | [^3^H]LSD (1 nM) | Alpha2C | [^3^H]Clonidine (1 nM) |
| D1 | [^3^H]SCH233930 (0.2 nM) | Beta1 | [^125^I]Iodopindolol (0.1 nM) |
| D2 | [^3^H]N-methylspiperone (0.2 nM) | Beta2 | [^125^I]Iodopindolol (0.1 nM) |
| D3 | [^3^H]N-methylspiperone (0.2 nM) | Beta3 | [^125^I]Iodopindolol (0.1 nM) |
| D4 | [^3^H]N-methylspiperone (0.2 nM) | M1 | [^3^H]QNB (0.5 nM) |
| D5 | [^3^H]SCH233930 (0.2 nM) | M2 | [^3^H]QNB (0.5 nM) |
| DOR | [^3^H]DADLE (0.3 nM) | M3 | [^3^H]QNB (0.5 nM) |
| KOR | [^3^H]U69593 (0.3 nM) | M4 | [^3^H]QNB (0.5 nM) |
| MOR | [^3^H]DAMGO (0.3 nM) | M5 | [^3^H]QNB (0.5 nM) |
| H1 | [^3^H]Pyrilamine (0.9 nM) | Sigma-1 | [^3^H]Pentazocine (3 nM) |
| H2 | [^3^H]Tiotidine (3 nM) | Sigma-2 | [^3^H]DTG (3 nM) |
| PBR | [^3^H]PK11195 (1 nM) |  |  |

Additional experimental details available in NIMH PDSP protocol book.[1]

1. Roth B. National Institute of Mental Health Psychoactive Drug Screening Program (NIMH PDSP) Assay Protocol Book Version II The University of North Carolina Chapel Hill. 2014 [cited 2014]. Available from: <https://pdspdb.unc.edu/pdspWeb/content/PDSP%20Protocols%20II%202013-03-28.pdf>.
